# Systemic Sclerosis-Related Intestinal Pseudo-Obstruction Mimicking Mechanical Small Bowel Obstruction

**DOI:** 10.7759/cureus.101784

**Published:** 2026-01-18

**Authors:** Anaan Fareed, Joel Petit

**Affiliations:** 1 General Surgery, John Hunter Hospital, Newcastle, AUS; 2 School of Medicine and Public Health, University of Newcastle, Newcastle, AUS

**Keywords:** acute intestinal pseudo-obstruction, scleroderma, small bowel dysmotility, small bowel obstruction, systemic sclerosis

## Abstract

Gastrointestinal involvement is common in systemic sclerosis (SSc); however, intestinal pseudo-obstruction represents a rare and severe manifestation that may closely mimic mechanical small bowel obstruction (SBO). Differentiating functional pseudo-obstruction from true mechanical obstruction remains challenging due to overlapping clinical and radiological features, and recurrent presentations despite exclusion of an anatomic cause are uncommon. We report a case of a 66-year-old man with limited cutaneous SSc who presented with recurrent episodes of abdominal pain, vomiting, distension, and obstipation over a 24-month period. Cross-sectional imaging during multiple admissions consistently demonstrated features suggestive of mechanical SBO, including small bowel dilatation, apparent transition points, fecalization, and ultimately a hide-bound appearance. Despite these findings, diagnostic laparoscopy revealed no mechanical obstruction. The patient experienced transient improvement with conservative management but had repeated re-presentations with progressively convincing radiological features. Management was further complicated by intolerance and limited response to multiple prokinetic agents. This case highlights an important diagnostic pitfall in which SSc-related intestinal pseudo-obstruction may present as a recurrent and progressively misleading radiological mimic of mechanical SBO, even after operative exclusion of a mechanical cause. Recognition of this entity is essential to avoid unnecessary surgical intervention and to facilitate appropriate conservative, nutritional, and multidisciplinary management. Clinicians should maintain a high index of suspicion for pseudo-obstruction in patients with SSc who present with recurrent obstructive symptoms, particularly when imaging findings are discordant with operative or clinical progression.

## Introduction

Systemic sclerosis (SSc) is a rare and heterogeneous autoimmune connective tissue disorder with the following subtypes: limited cutaneous systemic sclerosis (LcSSc) and diffuse cutaneous systemic sclerosis (DcScc) [1]. Gastrointestinal (GI) involvement occurs in 75-90% of SSc cases across both subtypes, and the small bowel is involved in 18-88% of the cases [1-3]. Unlike true mechanical small bowel obstruction (SBO), which results from a fixed intraluminal or extraluminal blockage, such as adhesions, hernia, or malignancy, intestinal pseudo-obstruction is characterized by severe gastrointestinal dysmotility causing functional obstruction in the absence of any mechanical occlusion. Intestinal pseudo-obstruction is a recognized but uncommon manifestation, occurring in approximately 3.7-5.4% of affected individuals, and represents the most severe end of the gastrointestinal dysmotility spectrum [4,5]. Although gastrointestinal involvement is frequent in SSc, only a minority of patients develop hypomotility profound enough to mimic mechanical SBO clinically.

The pathophysiology involves multiple mechanisms as follows: (1) autonomic neuropathy mediated by SSc-associated autoantibodies (anti-centromere, anti-Scl-70, and anti-muscarinic-3 receptor), (2) microvascular injury leading to ischemia, (3) progressive smooth muscle atrophy and replacement fibrosis [3,6,7]. These result in impaired peristalsis, intestinal stasis, and bacterial overgrowth, creating pseudo-obstructive symptoms [3,7].

Distinguishing SSc-related pseudo-obstruction from mechanical SBO is particularly challenging because both conditions present with abdominal pain, distension, nausea, and vomiting [8-10]. Radiologically, CT and MRI findings also overlap significantly; dilated small bowel loops, air-fluid levels, and even apparent transition points may be present in both [9,11-13]. As a result, in difficult or recurrent presentations, diagnostic laparoscopy is sometimes required to definitively exclude a mechanical cause [10].

Although SSc-related intestinal pseudo-obstruction has been reported, including the single-episode case described by Shah and Shahidullah, recurrent presentations that convincingly mimic mechanical SBO despite laparoscopic exclusion of mechanical cause remain rare [14]. As such, this report extends the existing literature by demonstrating that SSc-related pseudo-obstruction may present as a recurrent, progressively convincing radiological mimic of mechanical SBO, even after surgical exclusion of mechanical cause.

## Case presentation

A 66-year-old Caucasian man with established limited cutaneous systemic sclerosis (LcSSc) presented to our tertiary center in November 2023 with a one-week history of postprandial vomiting and generalized abdominal pain, accompanied by two days of obstipation. He reported a preceding six-month history, beginning in early 2023, of intermittent abdominal pain, nausea, vomiting, and constipation requiring regular laxative use. His LcSSc was diagnosed in 2020, with autoimmune serology demonstrating a positive anti-nuclear antibody (ANA) titre of 1:2560 with a nucleolar pattern, signifying a strong immune response, and an increased risk for cardiac and pulmonary involvement [15]. However, he had negative anti-Scl-70 and anti-centromere antibodies, and a perinuclear antineutrophil cytoplasmic antibodies (p-ANCA) titre of 1:80. He had experienced Raynaud’s phenomenon since 2013 and migratory polyarthralgia involving the shoulders, jaw, hips, and small joints of both hands. Additional gastrointestinal manifestations included gastroesophageal reflux disease and oropharyngeal dysphagia, for which prior upper gastrointestinal endoscopy revealed Los Angeles grade A reflux esophagitis and gastritis without other structural abnormalities. His surgical history was significant for an open appendicectomy at 17 years of age. He has a 40-pack-year smoking history, minimal alcohol consumption, and was independent with full functional capacity at baseline.

On examination, the patient was hemodynamically stable and afebrile, with a blood pressure of 105/70 mmHg, heart rate of 90 beats per minute, and temperature of 36.5°C. Abdominal examination demonstrated a soft but distended abdomen with periumbilical tenderness and no signs of peritonism. Cutaneous examination revealed skin thickening of the upper limbs, most pronounced at the distal fingers and extending proximally to just below the elbows bilaterally. Mild skin thickening was also noted over the forehead and maxillary regions. Telangiectasia was present on the palmar aspects of the hands, and Raynaud’s phenomenon was observed affecting the fingertips.

**Figure 1:**
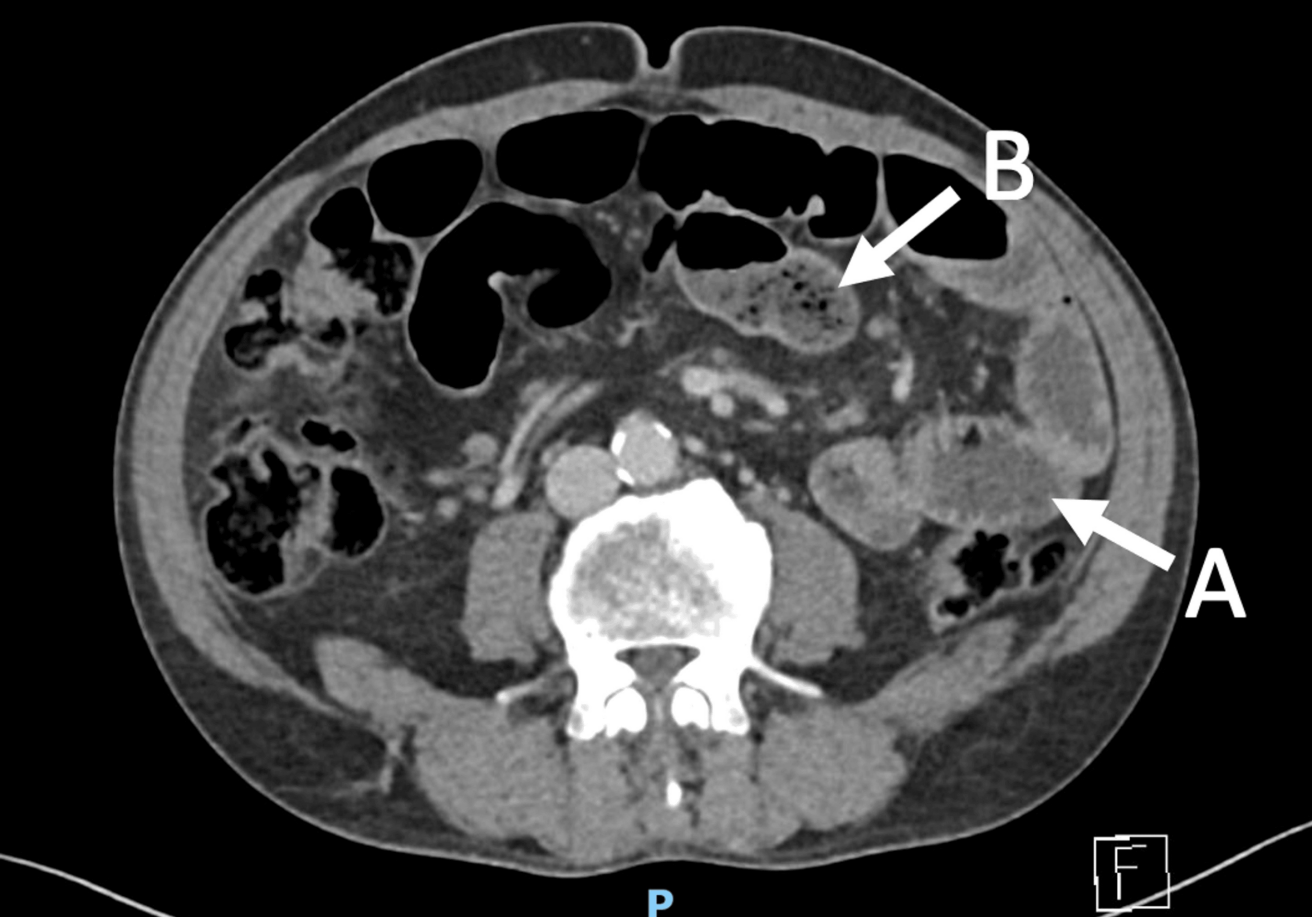
Axial view of CT scan. (A) Dilated small bowel loops and (B) small bowel fecalization and air-fluid levels.

Laboratory investigations demonstrated leukocytosis (white cell count: 13.7×10^9^/L) with a C-reactive protein of 2.4 mg/L. Renal function, electrolytes, and liver enzymes were within normal limits. Serum albumin was normal at 45 g/L. Thyroid function was normal with a TSH of 1.98 mIU/L. Nutritional indices, including vitamin B12 (218 pmol/L), folate (28.8 nmol/L), and ferritin (164 µg/L), were within normal ranges. Coagulation studies were unremarkable (international normalized ratio {INR}: 1.1, prothrombin time {PT}: 12 s, activated partial thromboplastin time {APTT}: 38 s). Fecal calprotectin was elevated at 204 µg/g. Serum immunoglobulins (IgA, IgG, and IgM) were within normal limits, and tissue transglutaminase antibodies were negative. A portal venous phase contrast-enhanced (Omnipaque-300) CT of the abdomen and pelvis, acquired with thin-slice imaging (0.6 mm), demonstrated multiple dilated loops of small bowel with gradual tapering to normal caliber in the left central abdomen. These findings were associated with small bowel fecalization and short segments of bowel wall thickening (Figure 1).

He was managed conservatively with bowel rest for 24 h and intravenous (IV) fluids (4 L of 0.9% sodium chloride and Hartmann's fluid over 24 h). His bowel movements resumed, and his symptoms of abdominal pain, nausea, and vomiting resolved. Oral intake was gradually reintroduced, and the patient was discharged after tolerating a diet.

**Figure 2:**
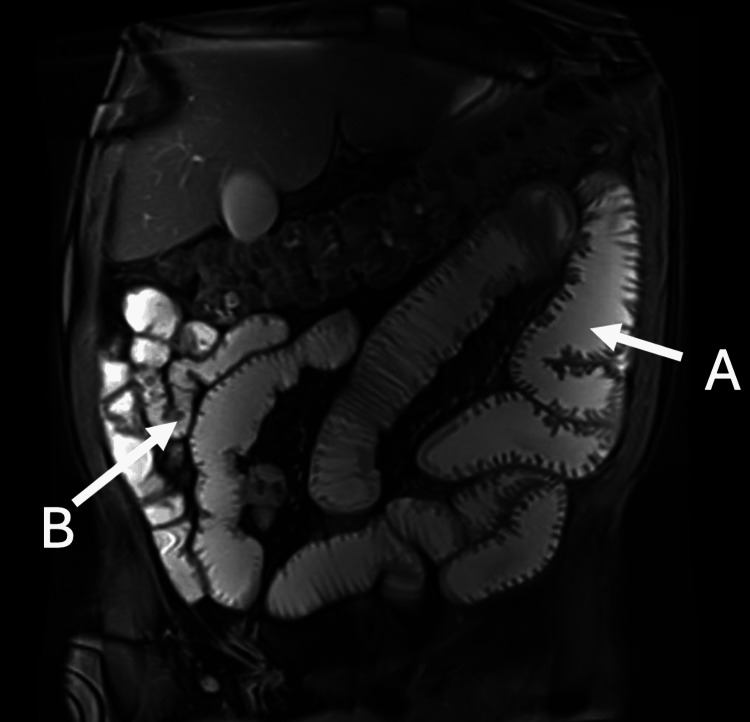
T2-weighted coronal view of MRI enterography. (A) Dilated proximal small bowel loops (B) apparent transition point with collapsed distal small bowel near the terminal ileum.

**Figure 3:**
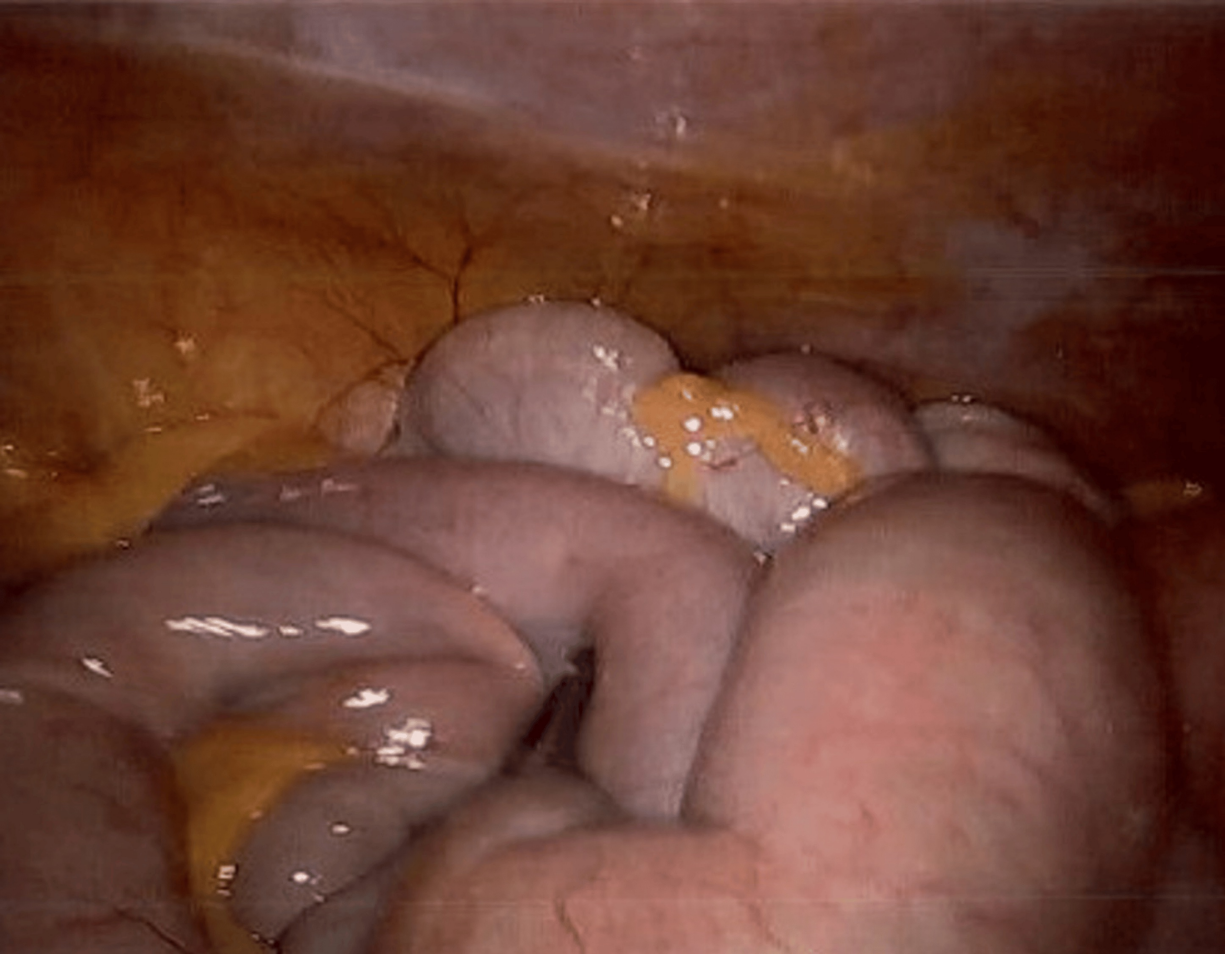
Intraoperative image during diagnostic laparoscopy showing normal looking small bowel loops.

Outpatient gadolinium-enhanced magnetic resonance enterography (MRE) in October 2023 demonstrated a subacute partial SBO with a possible transition point in the distal small bowel, where there was persistent collapse of downstream loops (Figure 2). Given the recurrent SBO-like presentations, persistent radiological suggestion of a transition point despite interval clinical improvement, and the inability to definitively exclude a mechanical cause on imaging alone, he subsequently underwent an elective diagnostic laparoscopy in April 2024 to exclude surgically correctable pathology. Intraoperatively, there were no significant adhesions, strictures, or stenoses. Patchy areas of small bowel serosal inflammation were observed; however, a complete run of the bowel from the terminal ileum to the duodenojejunal flexure demonstrated no dilatation or obstructing pathology (Figure 3).

He also underwent a colonoscopy in February 2025 with deep terminal ileal intubation to 15 cm and demonstrated mild erythema in the terminal ileum. Histopathological examination revealed focal mild neutrophilic infiltration of the surface epithelium, focal villous blunting, and increased lamina propria cellularity composed of lymphocytes, plasma cells, eosinophils, and occasional neutrophils, suggestive of mild chronic ileitis. Although histology demonstrated mild chronic ileitis, this was not considered causative of the recurrent obstructive episodes, as there were no endoscopic, radiological, or operative features to suggest Crohn’s disease, including strictures, deep ulceration, or skip lesions.

**Figure 4:**
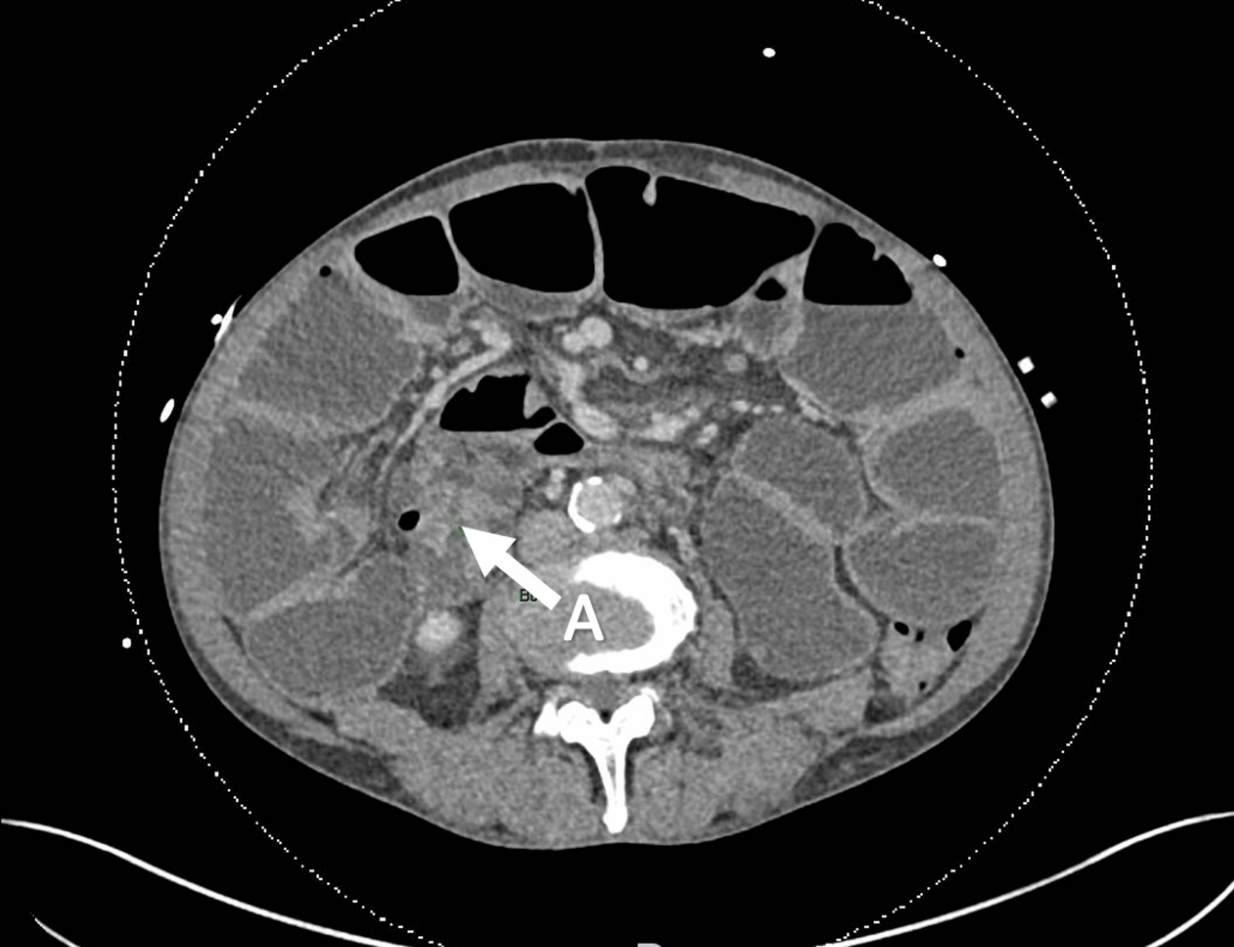
Admission 2 - axial view of CT scan showing apparent transition point at the distal terminal ileum. (A) Characterized by collapsed distal small bowel and tapering.

**Figure 5:**
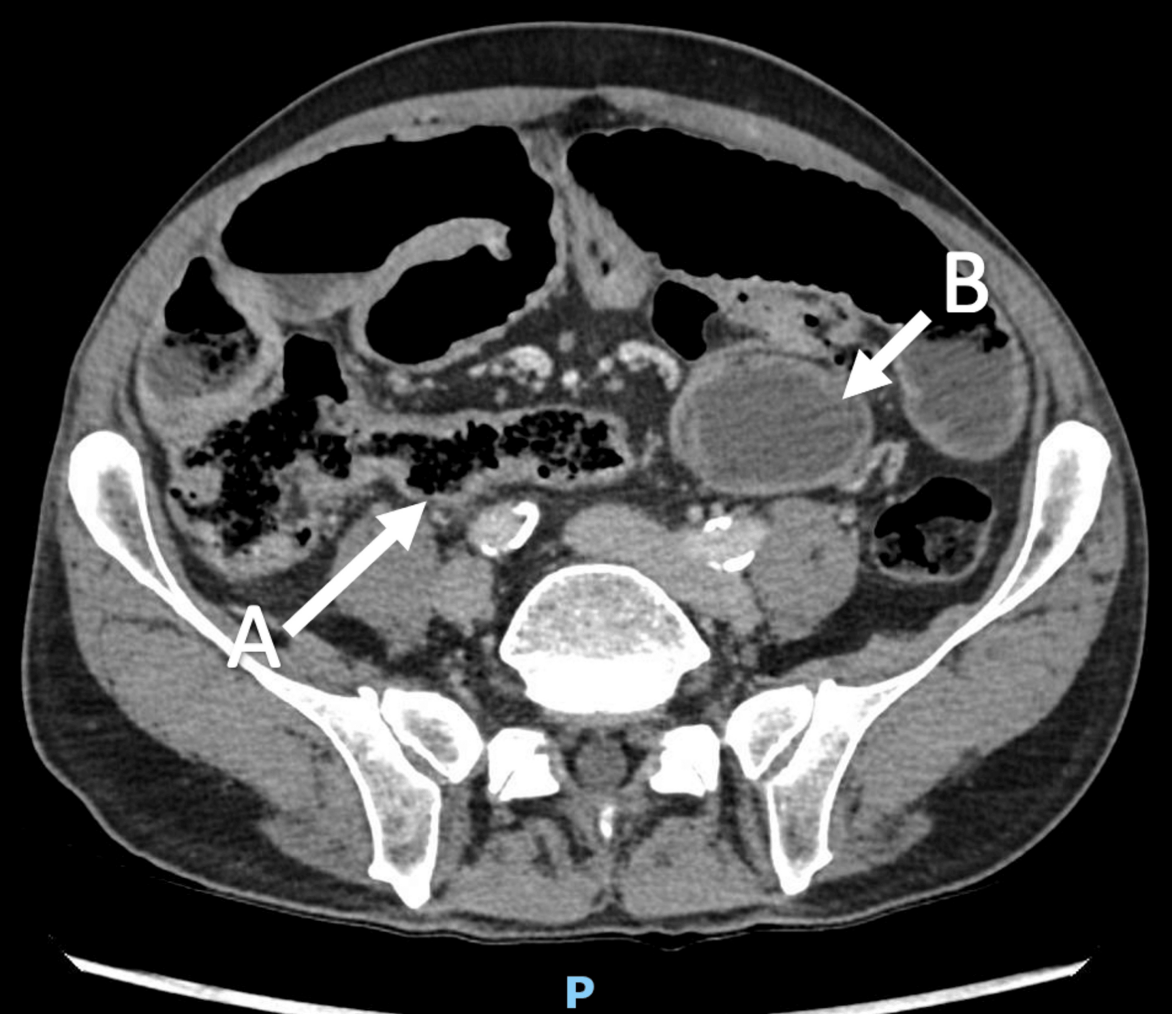
Admission 3 - (A) fecalization of terminal ileal content and (B) dilated proximal small bowel loop.

**Figure 6:**
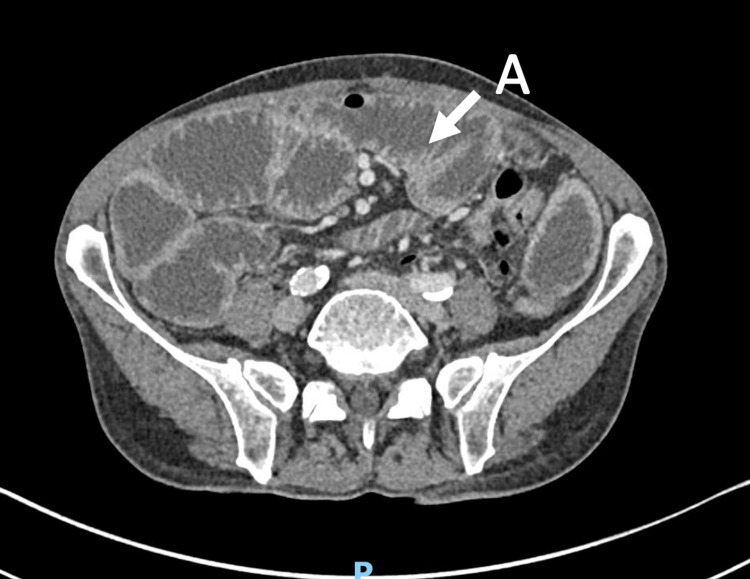
Admission 4 - hide-bound sign refers to the luminal dilation and decreased distance between adjacent valvulae conniventes. (A) Dilated small bowel loops with hide-bound sign.

Although outpatient antroduodenal manometry (ADM) was planned, it was not completed due to loss to follow-up. Additional functional studies, including formal colonic motility testing, were not performed. Following laparoscopy, the patient remained symptom-free for approximately two weeks before re-presenting with similar obstructive symptoms. Over the subsequent 24 months, he experienced three further hospital admissions with comparable clinical presentations. A summary of all four admissions is provided in Table 1. The CT images from the subsequent three admissions are shown in Figures 4-6.

**Table 1:** Summary of hospital admissions, CT findings, and management. NGT: nasogastric tube; TDS: three times daily

Admission	Date	Length of stay	CT findings	Management
1	October 2023	2 days	Dilated proximal small bowel with distal collapse, gradual transition point in left flank (Figure 1)	Nil by mouth for 24 h
				No NGT
				IV Hartmann's fluid and 0.9% saline at 100-150 mL/h (total 4 L over 24 h)
				Upgraded to a full diet after bowels opened
2	May 2024	3 days	Dilated small bowel loops with tapering at the distal ileum (Figure 4)	Nil by mouth for 48 h
				No NGT
				IV Hartmann's fluid at 60-200 mL/h (total 6 L over 72 h)
				Diet upgraded to full diet
				Planned for outpatient antroduodenal manometry but lost to follow-up
3	September 2024	2 days	Dilated small bowel loops to the terminal ileum, with distal gradual tapering, and fecalization of small bowel content (Figure 5)	Nil by mouth for 24 h
				No NGT
				IV Hartmann's fluid at 80 mL to 1 L/h (total 3 L over 24 h)
				IV metoclopramide 10 mg TDS, IV erythromycin 250 mg TDS
				Diet upgraded as tolerated and discharged
4	October 2025	4 days	Distended stomach, duodenum, and proximal small bowel with hide-bound sign in mid small bowel with transition point in terminal ileum with complete collapse distally (Figure 6)	NGT decompression with free drainage and 4-hourly aspirates
				Gastrografin follow-through study - 100 mL of Gastrografin given and NGT spigoted for 4 h postadministration, followed by free drainage and 4-hourly aspirates.
				Gastrografin seen in the transverse, descending, and sigmoid colon on abdominal X-ray at 10 h postadministration
				Diet upgraded as tolerated and discharged

The patient was reviewed on multiple occasions in the gastroenterology clinic between hospital admissions. A trial of domperidone at a dose of 10 mg three times daily was initiated in December 2024 and continued for six months, resulting in transient symptomatic improvement; however, this was discontinued due to persistent intermittent abdominal pain and bloating. In June 2025, he commenced on prucalopride 2 mg daily as per the treating gastroenterologist, which led to partial symptomatic improvement but was ceased in less than a week since commencement due to adverse reactions, including diaphoresis and vomiting.

Following his most recent admission in October 2025, the patient adopted small, frequent meals with oral nutritional supplementation (Ensure Plus twice daily) and remains under regular three-monthly dietetic review. He continues to take erythromycin 250 mg twice daily as a prokinetic agent. He was advised to recommence prucalopride at 1 mg daily for two weeks if tolerated and increase to 2 mg daily. He was mostly symptom-free but reported intermittent bloating and nausea, and remained functionally independent and at his baseline level of activity. At one-month follow-up after his last admission, he was reviewed in the gastroenterology clinic, with plans for repeat MRE and video capsule endoscopy. Ongoing follow-up was arranged with gastroenterology at three-month intervals, rheumatology every six months, and dietetics every three months. Overall, repeated exclusion of mechanical obstruction in the context of underlying systemic sclerosis raised suspicion for recurrent pseudo-obstruction related to intestinal dysmotility.

## Discussion

This case highlights a diagnostically challenging presentation of recurrent intestinal pseudo-obstruction in SSc, characterized by repeated episodes clinically and radiologically indistinguishable from mechanical small bowel obstruction despite exclusion of a mechanical cause on laparoscopy.

The pathophysiology of SSc-related pseudo-obstruction is complex, multifactorial, and incompletely understood. Proposed mechanisms include autonomic neuropathy mediated by SSc-associated autoantibodies, microvascular injury, and progressive smooth muscle atrophy and fibrosis, ultimately resulting in impaired peristalsis, intestinal stasis, bacterial overgrowth, and small bowel dilatation [3,6,7,16]. In addition to anti-centromere and anti-Scl-70 antibodies, antibodies targeting muscarinic-3 receptors also play a role in pseudo-obstruction [7,17]. These changes create a clinical picture that may be challenging to distinguish from true mechanical obstruction, with recurrent episodes of abdominal pain, distension, vomiting, and obstipation [10].

A major challenge in SSc-related pseudo-obstruction is its substantial radiological overlap with mechanical SBO. While plain abdominal radiography was historically used, CT has become the primary imaging modality for evaluating suspected obstruction, with reported sensitivities of 91-94% and specificities of 88-96% [12,13]. CT features suggestive of mechanical obstruction include small bowel dilatation (≥3 cm), a transition point between dilated and collapsed bowel, air-fluid levels, fecalization of small bowel contents, and a decompressed colon [9,12,13]. Similar findings, however, may also be present in intestinal pseudo-obstruction, including bowel dilatation and air-fluid levels [10]. In addition, characteristic radiographic signs of SSc, such as the "hide-bound sign," defined by markedly dilated small bowel with closely approximated and thickened valvulae conniventes, have been described [18]. Even in the absence of a true mechanical blockage, some patients, including the case presented here, may exhibit features such as apparent transition points or distal tapering that can closely mimic fixed obstruction and contribute to diagnostic uncertainty. This was evident in our patient, whose CT and magnetic resonance enterography (MRE) repeatedly suggested a transition point despite the absence of mechanical obstruction on laparoscopy.

The differential diagnosis of intestinal pseudo-obstruction is broad and includes medication-induced causes, mitochondrial disorders (such as mitochondrial neurogastrointestinal encephalomyopathy), neuromuscular and autonomic disorders (including Duchenne muscular dystrophy and multiple system atrophy), and infiltrative diseases such as amyloidosis [19]. Other autoimmune and connective tissue disorders, including celiac disease, systemic lupus erythematosus, anti-synthetase syndrome, and inflammatory myopathies, should also be considered and appropriately investigated [19]. From a surgical perspective, adhesive disease, occult internal hernia, and early or fibrostenotic Crohn’s disease may closely mimic intestinal pseudo-obstruction, particularly in patients with prior abdominal surgery or histological ileitis. In this case, these were considered but deemed unlikely given the absence of stricturing or transmural inflammation on endoscopy, non-progressive imaging findings, and exclusion of obstructing pathology on laparoscopy.

Before establishing a diagnosis of pseudo-obstruction, exclusion of mechanical SBO remains essential. In cases where imaging findings are inconclusive, diagnostic laparoscopy may be warranted to exclude obstructive pathology such as adhesions or strictures [10]. Antroduodenal manometry (ADM) is considered the diagnostic gold standard for evaluating gastrointestinal motility disorders, including pseudo-obstruction [16,20]. However, it is invasive, time-consuming (lasting up to 6 h for stationary testing or 24 h for ambulatory studies), and often poorly tolerated by patients. Consequently, ADM is infrequently performed in routine practice, with only approximately 21% of clinicians specializing in severe gastrointestinal dysmotility reporting its use in more than half of cases [20,21]. The absence of formal motility testing in this case represents an important diagnostic limitation, as ADM could have provided objective confirmation of dysmotility and helped distinguish neuropathic from myopathic patterns of gastrointestinal involvement. Without manometric data, a degree of diagnostic uncertainty remains, and alternative causes of pseudo-obstruction cannot be entirely excluded. Nevertheless, the diagnosis of SSc-related pseudo-obstruction is supported by the patient’s established systemic sclerosis, recurrent stereotyped presentations, exclusion of mechanical obstruction on laparoscopy, and imaging features consistent with severe dysmotility. When performed, ADM can identify characteristic abnormalities, such as absent migrating motor complexes, low-amplitude contractions, impaired antral-duodenal coordination, and absent fed responses, and may facilitate assessment of pharmacologic responses to prokinetic agents administered during testing [20]. Capsule endoscopy is another investigation that can assess small bowel transit time and is planned for our patient in this case [16,20]. Other modalities, such as barium studies, may also be informative but are now rarely employed in contemporary practice [16].

Mecoli et al. reported that approximately 70% of patients with SSc-related intestinal pseudo-obstruction improved with supportive management, including bowel rest, nasogastric decompression, fluid resuscitation, and correction of electrolyte abnormalities, and noted that recurrent episodes were associated with greater nutritional compromise and poorer outcomes [5]. In contrast, despite initial resolution with conservative measures, our patient experienced four recurrent hospital admissions, each separated by symptom-free intervals. The complexity of management in this case is likely multifactorial and may reflect ongoing risk factors, such as continued smoking and intolerance or limited response to multiple prokinetic agents. A limitation of this report is the incomplete diagnostic evaluation, as formal motility studies were not performed; however, further assessment with video capsule endoscopy is planned. Importantly, population-level data from Valenzuela et al. demonstrate that SSc-related pseudo-obstruction is associated with longer hospital stays, increased reliance on parenteral nutrition, and an in-hospital mortality rate of 7.3%, underscoring the clinical significance of recurrent disease [4].

Prokinetic agents can be used for symptomatic relief in SSc-related pseudo-obstruction; however, the evidence supporting their efficacy remains limited and heterogeneous. Dopamine-2 receptor antagonists, such as metoclopramide and domperidone, exert prokinetic effects predominantly on gastric motility, with more modest activity in the small bowel, and their use is generally restricted to short-term therapy because of adverse effects [22]. Erythromycin, a macrolide antibiotic with motilin receptor agonist activity, has also been utilized; however, its benefit in SSc-associated dysmotility appears limited, with some studies suggesting paradoxical inhibition of intestinal motility [3,22,23]. Prucalopride, a selective serotonin (5-HT₄) receptor agonist originally developed for chronic constipation, has demonstrated whole-gut prokinetic properties and has been associated with improvements in symptoms such as abdominal pain and bloating in intestinal pseudo-obstruction [22,24]. Octreotide, a somatostatin analog, has also been shown to improve small bowel motility in selected cases; however, its use may be limited by delayed gastric emptying and worsened constipation, which can exacerbate symptoms in some patients [3,7].

Nutritional support is a pivotal component of management in patients with SSc-related gastrointestinal dysmotility and should be guided by a specialist dietician's input. Dietary strategies include small, frequent, soft meals supplemented with high-protein and energy-dense oral nutritional products [16]. Monitoring for and replacement of micronutrient deficiencies, including fat-soluble vitamins, vitamin B12, and iron, may be necessary [16]. In patients unable to maintain adequate oral intake or at significant risk of malnutrition, total parenteral nutrition or home parenteral nutrition should be considered. Optimal management of gastrointestinal manifestations in SSc requires a multidisciplinary approach involving gastroenterologists, dietitians, rheumatologists, and surgeons when appropriate [16].

## Conclusions

SSc-related intestinal pseudo-obstruction is an uncommon but clinically significant manifestation that can closely and repeatedly mimic mechanical small bowel obstruction. This case highlights the diagnostic challenges posed by recurrent presentations with progressively convincing radiological features despite repeated exclusion of a mechanical cause on laparoscopy. Recognition of this entity is essential to avoid unnecessary surgical intervention and to prompt appropriate conservative, nutritional, and multidisciplinary management. Clinicians should maintain a high index of suspicion for pseudo-obstruction in patients with SSc who present with recurrent obstructive symptoms, particularly when imaging findings are discordant with operative or clinical progression. Early identification and coordinated care are crucial, as recurrent disease is associated with substantial morbidity, nutritional compromise, and prolonged hospitalization.
